# The Medical Society

**Published:** 1896-10-31

**Authors:** 


### THE MEDICAL SOCIETY.

At the meeting held on October 26th, Mr. P. J. Freyer discussed "The Best Method of Removing Large Vesical Calculi," strongly advocating the employment of litholapaxy for the purpose. Among the last 300 cases that he had had to do with, the ages of the patients ranging up to ninety years, and the weight of the stones to six and eight ounces, he had only performed a cutting operation seven times. It was impossible to draw a hard and fast line as to what was meant by a large stone, the age of the patient and the experience of the operator being points of great importance. But taking the question only as it referred to adults, he would class all stones weighing two ounces and upwards as large stones. In his own practice, out of nearly 900 stones operated on by all methods, 49 cases fell within this definition. In selecting an operation for such cases it must be remembered that largeness of the stone usually connoted other things, especially long duration, so that most patients so affected were in bad health at the time of the operation; cystitis, ulcered bladder, and fetid urine occurring in many cases, and the kidneys being affected in the majority. Some of his cases were also suffering from diarrhœa, dysentery, and hypertrophy of the spleen. Of these forty-nine cases, in four the stones were removed by suprapubic operation, thirteen by lateral lithotomy, one through the vagina, and thirty-one by litholapaxy. He had employed the suprapubic method in the case of the largest stone he had removed. It weighed twelve and a half ounces, and the patient died. He thought that where there was a stricture, and the stone was small, the stricture might be cut, and litholapaxy employed, but in large stones it would be best to do lithotomy. Again, if the prostate were greatly enlarged, lithotomy was required, although moderate enlargement was no bar to litholapaxy, and there were certain cases in which the bladder was so tightly contracted on the stone that it was impossible to introduce and work the lithotrite, and these again would have to be cut. But otherwise litholapaxy was the operation. The discussion which follow turned chiefly on the use of Petersen's bag, the general feeling seeming to be that it was not of any essential service in cases of stone, although it was of considerable assistance in exploring the base of the bladder.

